# Lessons From Mapping the Structural and Social Determinants of Health for Older Adults Living in NYC Chinatowns

**DOI:** 10.1093/geroni/igaf122.4152

**Published:** 2025-12-31

**Authors:** Isadora Li, Dalilah Mora, Paris Adkins-Jackson

**Affiliations:** Barnard College Department of Political Science, New York, New York, United States; Columbia Mailman School of Public Health, Bronx, New York, United States; Mailman School of Public Health, Columbia University, New York, New York, United States

## Abstract

Older adults racialized as Asian are an underrepresented group in health disparities research. Neighborhoods commonly referred to as Chinatowns in NYC have older adult populations in which the majority are racialized as Asian and who face unique challenges to healthy aging due to structural determinants of health. This study aimed to compile a comprehensive directory of health resources in three New York City Chinatowns (Manhattan Chinatown, Flushing, and Sunset Park) in order to understand the landscape of health access for older adults in these neighborhoods. The study used a modified approach to community asset mapping, which involved teams of researchers conducting walking surveys within the target neighborhoods. Community asset mapping is a proven method of identifying community resources, but challenges arise when older adults are engaged in this approach due to mobility limitations. The protocol yielded a novel dataset of 1,154 resources (313 in Sunset Park, 364in Manhattan, 477 in Flushing) among four broad categories: healthcare, older adults, social services, and health promotion. The study also produced three neighborhood-specific Google Maps albums of determinants. The team then collaborated with a local organization to further refine a portion of the dataset to align with their efforts to support older adults living in an affordable senior housing building. This project’s approach to asset mapping has implications for generating methods of investigation involving older adults which accommodate their needs, such as accommodations due to mobility limitations. The novel dataset and collection of maps will also drive continued work with community organizations.

